# Influence of Frailty Status on the Efficacy of Epidural Steroid Injections in Elderly Patients With Degenerative Lumbar Spinal Disease

**DOI:** 10.1155/2024/5038496

**Published:** 2024-09-07

**Authors:** Hee Jung Kim, Ho Jae Nam, Shin Hyung Kim

**Affiliations:** Department of Anesthesiology and Pain Medicine Anesthesia and Pain Research Institute Yonsei University College of Medicine, Seoul, Republic of Korea

**Keywords:** degenerative lumbar spinal disease, elderly patients, epidural steroid injection, frailty

## Abstract

**Background:** The global increase in the elderly population has led to a higher prevalence of degenerative lumbar spinal diseases. Epidural steroid injection (ESI) is a widely used procedure for managing lower back pain. This study investigated the association of preprocedural frailty status with the efficacy of ESI in elderly patients diagnosed with degenerative lumbar spinal diseases.

**Methods:** This retrospective observational study included patients aged 65 years and older who underwent lumbar ESI. Frailty status (robust, prefrail, and frail) assessed via the Frailty Phenotype Questionnaire was collected along with demographic and clinical parameters. Good analgesia was defined as a ≥ 50% reduction in pain score at 4-week follow-up evaluation. Multivariable logistic regression analyses were performed to identify factors associated with poor analgesia.

**Results:** We included 289 patients in this study. Frailty status correlated with analgesic outcomes, with worsening frailty status correlating with increasingly poor analgesia after the injection (robust = 34.5%, prefrail = 40.8%, and frail = 60.0%, *p*=0.003), predominantly in female patients. After adjusting for demographic and clinical factors, frail patients demonstrated much higher odds of poor analgesia than robust individuals (adjusted odds ratio [aOR] = 2.673, 95% confidence interval [CI] = 1.338–5.342, *p*=0.005). Conversely, prefrail patients did not show a significant association with analgesic outcome (aOR = 1.293, 95% CI = 0.736–2.272, *p*=0.372).

**Conclusions:** Frailty, but not prefrailty, appeared to be an independent factor associated with poor analgesic efficacy of ESI in elderly patients with symptomatic degenerative lumbar spinal disease receiving conservative care.

## 1. Introduction

The global elderly population is experiencing rapid growth, driven by socioeconomic progress and significant medical advancements [1]. This demographic shift contributes to an increased prevalence of chronic low back pain [2], with degenerative lumbar spinal diseases constituting a significant portion of radiographic pathologies in this population [3]. Among the numerous treatment options for relieving pain in older individuals experiencing chronic low back pain, epidural steroid injection (ESI) is one of the most frequently performed procedures [4, 5]. Several prognostic factors have been proposed to predict pain relief following ESI, including the grade of nerve root compression by magnetic resonance imaging (MRI), interferon-gamma levels from epidural lavage fluid, and epidural contrast dispersal patterns during the procedure [6, 7]. Additionally, a prior investigation [8] revealed that reduced handgrip strength (HGS), a key diagnostic factor for sarcopenia [9], was independently associated with poor analgesic efficacy of ESI in elderly patients with low back pain.

Frailty is characterized by a deterioration in functioning across various physiological systems, coupled with a heightened susceptibility to stressors [10, 11]. Frailty is a significant risk factor for mortality in elderly people and is associated with a diverse array of outcomes, including falls, disability, worsening mobility, fractures, depression, cognitive decline, and hospitalization [11]. Additionally, across different spinal surgery populations, frailty has been reported as a predictor of adverse events, mortality within various timespans, hospital length of stay, and discharge disposition [12, 13]. However, no previous study has explored the relationship between frailty status and the analgesic effects of ESI in elderly individuals with degenerative lumbar spinal disease.

Therefore, our objective in this study was to assess the association between preprocedural frailty status and the analgesic efficacy of ESI in elderly patients with degenerative lumbar spinal disease. Additionally, we aimed to determine whether frailty status would be independently associated with the analgesic efficacy of ESI in this population.

## 2. Methods

### 2.1. Study Population

**Figure 1:**
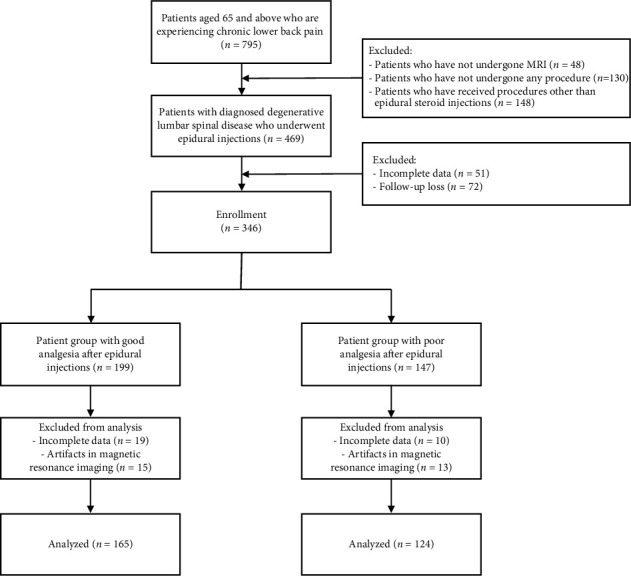
Study flowchart.

This study was approved by the Institutional Review Board of Yonsei University Health System in Seoul, Republic of Korea (no. 4-2023-1346). Given the retrospective observational design of this study, the requirement for obtaining informed consent from patients was waived. The study's flowchart is depicted in Figure 1. Among patients aged 65 years and older who visited our pain clinic between March 2022 and February 2023, we included those who presented with degenerative lumbar spinal disease and received a lumbar ESI. Patients who did not undergo an MRI examination and those who did not receive ESI or underwent procedures other than ESI were excluded. Additionally, patients with no follow-up or incomplete electronic medical records and patients with artificial shadows on their MRI images due to surgical instruments were also excluded from this study.

### 2.2. Screening for Frailty

As a frailty screen, the Frailty Phenotype Questionnaire [14] was administered during the initial outpatient clinic visit. This questionnaire consists of five components: fatigue, resistance, ambulation, inactivity, and weight loss.

1. Fatigue: Participants were assigned 1 point if they reported feeling like everything they do is an effort for more than 3 days a week. They received 0 points if they reported this feeling for fewer than 3 days a week.
2. Resistance: Participants received 1 point if they reported difficulty ascending 10 stairs without assistance and 0 points if they reported no difficulty.
3. Ambulation: Participants received 0 points if they reported no difficulty walking around a 400-m playground track. One point was awarded if they reported any level of difficulty, such as “a bit,” “very,” or “unable to do it at all.”
4. Inactivity: Participants received 0 points if they reported engaging in moderate or vigorous physical activity at least once in the past 7 days. One point was assigned if they answered “no,” indicating no such activity. Moderate-intensity physical activity included tasks such as fast walking, carrying light objects, cleaning, and infant care, and vigorous physical activity encompassed activities such as lifting heavy objects, digging, construction labor, and carrying objects upstairs.
5. Loss of weight: One point was assigned for the occurrence of unintentional weight loss of 4.5 kg or more within the past year.

According to the Fried frailty phenotype criteria, participants scoring “0” on the Frailty Phenotype Questionnaire were categorized as *robust*, those with a score of 1 or 2 were designated as *prefrail*, and individuals with a score of 3 or more were identified as *frail*.

### 2.3. Fluoroscopy-Guided Lumbar ESI

All procedures were conducted by two practitioners with similar clinical experience. Patients were positioned in the prone posture and sterilely draped for the procedure. Local anesthesia with 1% lidocaine was applied to the skin and soft tissue in the targeted needle entry area. For the interlaminar approach, a 20-gauge, 10-cm Tuohy needle was inserted into the target interlaminar space with anteroposterior fluoroscopic guidance. The loss of resistance technique was employed to navigate the needle tip accurately to the epidural space. Upon achieving loss of resistance, a lateral view was taken to ensure the precise placement of the needle tip. In the transforaminal approach, under an oblique view, a 22-gauge, 10-cm Quincke tip needle was inserted and cautiously advanced just caudad to the inferior margin of the pedicle. With a lateral view, the needle was further progressed into the neural foramen, immediately superior, lateral, and anterior to the exiting nerve root, until it encountered the anterior epidural space [15]. In the caudal approach, a 22-gauge, 8-cm Quincke tip needle was inserted just below the sacral hiatus at a 45° angle, reaching the bone. After a slight withdrawal and repositioning parallel to the skin, it was advanced into the sacral canal. Confirmation of the needle's placement in the caudal epidural space was done using a lateral fluoroscopic view. In all injections, to ensure appropriate epidural spread, 1-2 mL of contrast medium was administered during each injection. For the interlaminar approach, 5 mL of 0.5% lidocaine mixed with 5 mg of dexamethasone and 1500 IU of hyaluronidase was injected. For each level of the transforaminal approach, 2 mL of 0.5% lidocaine mixed with 5 mg of dexamethasone and 1500 IU of hyaluronidase was administered. The caudal approach involved injecting 15 mL of 0.2% lidocaine mixed with 5 mg of dexamethasone and 1500 IU of hyaluronidase.

### 2.4. Patient Demographics and Clinical Data Measurements

Demographic and clinical data were extracted through a review of electronic medical records. HGS was measured according to the standard protocol during the initial visit [8]. The collected patient characteristics included age, sex, body mass index (BMI), diagnosed comorbidities with current medications (hypertension, diabetes mellitus, and cardiovascular disease), history of cancer, psychological disease, osteopenia/osteoporosis, and prior spinal surgery. Pain-related factors included the duration of pain, baseline numeric rating scale, opioid usage for at least 1 month before ESI, and the presence of sleep disturbance. MRI findings were collected to assess the presence of a herniated disc, graded foraminal stenosis, or central stenosis [16, 17], compression fracture, and spondylolisthesis. The method of epidural injection—interlaminar, transforaminal, or caudal—was identified in the study population. Additionally, patients progressing to surgery within 1 year after ESI were investigated. For this study, good analgesia was defined as a ≥ 50% decrease in pain score at 4 weeks after the procedure without an increase in analgesic medication [18]. Conversely, poor analgesia was defined as a < 50% decrease in pain score at 4 weeks after the procedure.

### 2.5. Data Analysis

All statistical analyses were performed using the Statistical Package for the Social Sciences, version 26.0 (IBM Corp, Armonk, NY, USA). Descriptive statistics are expressed as the mean ± standard deviation for continuous variables and numbers (percentages) for categorical variables. Ordinal data and non-normally distributed continuous variables are presented as the median and interquartile range, and the normality of the data was evaluated using the Shapiro–Wilk test. Demographic and clinical parameters were compared using unpaired Student's *t*-tests, *χ*^2^ tests, or Fisher's exact tests, as appropriate. The Mann–Whitney U test was applied for non-normally distributed continuous variables. The trend for the percentage of patients with poor analgesia after ESI according to frailty status was analyzed using the chi-square test for linear-by-linear association. Because some independent variables in the model have the potential for high correlations, multicollinearity in the regression model was evaluated using variance inflation factors. Multivariable logistic regression analyses using backward elimination with likelihood ratio tests were conducted to identify predictors of poor analgesia following lumbar ESI, resulting in adjusted odds ratios (aORs) and corresponding 95% confidence intervals (CIs). A *p* value less than 0.05 was considered statistically significant.

## 3. Results

Throughout the study duration, 469 patients older than 65 years were diagnosed with degenerative lumbar spinal disease and underwent ESI. Among them, 289 patients were included in the study after excluding those who met the exclusion criteria (Figure 1).

**Figure 2:**
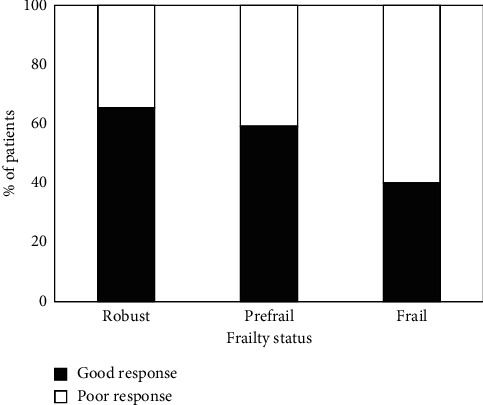
The distribution of patients with good (■) and poor (□) analgesia 4 weeks after epidural steroid injection categorized by frailty status.

Table 1 compares patient characteristics and clinical data between individuals who experienced good analgesia and those with poor analgesia after ESI. No significant differences were observed between the groups in terms of age, sex, BMI, and medical comorbidities (hypertension, diabetes mellitus, cardiovascular disease, cancer, psychological disease, osteoporosis/osteopenia, and prior lumbar surgery). The two groups had similarities in their pain-related data: pain duration, baseline pain score, opioid use history, and sleep disturbance. The MRI findings before the procedure revealed no significant differences between the groups. However, the HGS in the good analgesic group was significantly higher than the HGS in the poor analgesic group (24.96 ± 10.29 vs. 21.11 ± 8.53, *p*=0.001). Additionally, a notable difference was observed between groups categorized based on frailty status. The percentage of patients experiencing poor analgesia 4 weeks post-ESI increased significantly with worsening frailty status (robust = 34.5%, prefrail = 40.8%, and frail = 60.0%, *p*=0.003) (Figure 2).

Table 2 compares the frailty status of patients between the good and poor analgesia groups according to sex. Among male patients, the deterioration of frailty status had some correlation with an increase in the number of individuals experiencing poor analgesia following ESI; however, no significant difference was observed among the three groups (robust = 35.1%, prefrail = 42.2%, and frail = 57.7%, *p*=0.083). In contrast, among female patients, the number of individuals with poor analgesic effects rose substantially as frailty status worsened (robust = 34.0%, prefrail = 40.2%, and frail = 61.8%, *p*=0.016).

We found no multicollinearity among the independent variables. After adjusting for demographic and clinical data, the results of the multivariable logistic regression analyses (Table 3) demonstrate that a low HGS value (aOR = 0.959, 95% CI = 0.933–0.985, *p*=0.002) was an independent predictor of poor analgesia after lumbar ESI. Frail patients exhibited 2.673 times higher odds of poor analgesia after ESI than robust individuals (aOR = 2.673, 95% CI = 1.338–5.342, *p*=0.005). In contrast, prefrailty did not show a significant association with analgesic efficacy after ESI (aOR = 1.293, 95% CI = 0.736–2.272, *p* = 0.372).

## 4. Discussion

In this study, we aimed to determine whether frailty status would be associated with the analgesic efficacy following ESI in elderly patients with chronic low back pain with or without lumbar radicular pain. Our findings showed that a significant number of frail patients experienced poor analgesia after ESI. Therefore, preprocedural frailty status is an important factor for predicting the analgesic efficacy of ESI in this population.

As the most effective indicator of age-related muscle changes, reduced muscle strength is a crucial factor for diagnosing sarcopenia and is linked to physical disability in completing instrumental activities of daily living and functional limitations [19]. Even though the relationship between sarcopenia and frailty has not been fully characterized, these conditions are recognized to share many similar clinical outcomes and a proposed pathophysiology [20]. Moreover, the defining criteria of the frailty phenotype and sarcopenia have significant overlap [9, 21]. Thus, sarcopenia is integral to the manifestation of physical frailty, and the coexistence of physical frailty and sarcopenia is evident [10]. In a previous study [8], we demonstrated the association between HGS as a measure of sarcopenia and the analgesic effect of ESI. Similarly, the present study found comparable results regarding the association between frailty status, which might be considered a manifestation of sarcopenia, and the analgesic effect of ESI. These consistent findings imply that HGS and frailty status are closely related and that both might serve as important predictive factors for the analgesic effect of ESI.

In previous studies [22–25], frailty was closely linked to chronic low back pain and unfavorable outcomes related to spinal surgery, such as postoperative pain and complications. Similarly, our study revealed that frail patients are associated with experiencing poorer analgesic outcomes following spine-related procedures compared with their robust elderly counterparts, with the odds almost three times higher. Thus, our findings underscore the significance of considering frailty as a crucial variable in predicting pain outcomes in elderly patients with degenerative spinal disease.

What makes this study intriguing is the observed sex difference in the association between analgesic efficacy after ESI and frailty. There is insufficient information about sex differences in the relationship between frailty and responses to pain interventions. Frailty and its progression result from a combination of multidomain influences, including biological, social, and behavioral factors [26]. A study that used large population data to examine community-dwelling older adults found a higher prevalence of frailty in women than in men [27]. Additionally, an earlier report suggested that the influence of paraspinal fat infiltration on the analgesic effectiveness of ESI might be particularly pronounced in elderly female patients [28]. Skeletal muscle undergoes age-related changes that involve alterations in its architecture and macronutrient metabolism [29]. Notably, the decline in resting energy expenditure occurs more rapidly in both skeletal muscle and overall adipose tissue in females than in males [30]. This difference might contribute, in part, to the higher susceptibility of females to frailty compared with males. This sex-specific variation adds a nuanced layer to the findings, emphasizing the importance of considering both frailty and sex when evaluating analgesic outcomes.

Recent research has demonstrated that frailty is a dynamic process [31]. Across various studies, it was consistently observed that both deterioration and improvement in frailty were prevalent. During a 4.5-year investigation involving 754 community residents aged 70 years and older in the United States, 58% of participants underwent at least one transition related to frailty [32]. In a longitudinal study conducted in Ireland, individuals categorized as prefrail exhibited a 32% probability of reversing to a robust state. Additionally, those classified as frail had an 18% probability of reverting to a prefrail condition [33]. Those findings have implications for clinical practice. They underscore the significance of early frailty identification and emphasize the need for timely interventions. Furthermore, incorporating regular frailty assessments into routine clinical care for elderly adults is crucial for monitoring frailty changes. Recognizing when an individual reaches a specific frailty level is essential to trigger evidence-based clinical actions that address their unique needs [11, 26, 31]. In this study, frailty emerged as a reliable factor for predicting ESI outcomes compared with robust elderly individuals, whereas prefrailty did not exhibit the same predictive capacity. Thus, this result implies that the detection, management, and prevention of frailty could positively influence the efficacy of ESI in this population.

Our study is subject to several limitations. First, its cross-sectional nature prevents the establishment of a causal relationship between the efficacy of ESI and frailty. Additionally, as a retrospective study, the presence of selection bias and information bias cannot be ruled out. Moreover, the study's scope is confined to elderly Korean participants recruited from healthcare settings, potentially limiting the generalizability of the findings to a broader elderly population. The study's reliance on a real-world clinical practice model, in which physicians determine the timing for ESI or analgesic use, adds an element of variability that could affect the study's conclusions. In addition, even if the pain reduction is less than 50%, it can be considered good analgesia if the use of painkillers has decreased. However, this was not known since the use of painkillers other than opioids was not investigated in this study. The uniqueness of spinal disease introduces another layer of complexity because the disease itself might significantly influence frailty measures. Symptoms such as difficulty ascending stairs, walking challenges, and inactivity, all characteristic of lumbar spinal stenosis, contribute to the diagnosis of frailty [13]. ESI might itself serve as an effective intervention for phenotypic frailty by potentially reversing those symptoms. Although currently in the realm of theory, exploring postprocedural clinical data, including changes in frailty, is a concept that merits deeper investigation.

## 5. Conclusions

In summary, our study has demonstrated that frailty, but not prefrailty, was significantly associated with poor analgesia after ESI in elderly patients with degenerative lumbar spinal disease. This finding underscores the importance of screening for frailty status as one way to predict the analgesic efficacy of ESI and indicates the need for an interventional study to determine whether reverting to a prefrail condition can improve the analgesic efficacy of ESI in frail patients.

## Figures and Tables

**Table 1:** Patient characteristics and clinical data of individuals who experienced good analgesia and those who had poor analgesia after an ESI.

**Variables**	**Good analgesia (*n* = 165)**	**Poor analgesia (*n* = 124)**	***p* value**
Patient characteristics			
Age, years	74.22 ± 6.11 (65–96)	72.88 ± 5.55 (65–88)	0.055
Female sex	104 (63.0)	77 (62.1)	0.871
BMI, kg/m^2^	22.96 (21.10; 25.24)	23.22 (20.88; 25.23)	0.927
Medical comorbidities, *n*	46 (27.9)	34 (27.4)	0.931
Cancer, *n*	18 (10.9)	14 (11.3)	0.919
Psychological disease, *n*	20 (12.1)	14 (11.3)	0.828
Osteopenia/osteoporosis, *n*	20 (12.1)	12 (9.7)	0.512
Prior lumbar surgeries, *n*	38 (23.0)	39 (31.5)	0.109
Handgrip strength, kg	24.96 ± 10.29	21.11 ± 8.53	0.001
Pain-related data			
Pain duration, months	8.00 (0.25–240.00)	12.00 (0.25–240.00)	0.186
Baseline pain score, NRS 0–10	6.73 ± 1.74	6.90 ± 1.79	0.423
Opioid usage, *n*	57 (34.5)	38 (30.6)	0.485
Sleep disturbance, *n*	56 (33.9)	45 (36.3)	0.678
Preprocedural MRI findings, *n*			
Herniated disc	132 (80.0)	95 (76.6)	0.488
Foraminal stenosis			0.825
None to mild	86 (52.1)	63 (50.8)	
Moderate to severe	79 (47.9)	61 (49.2)	
Central stenosis			0.598
None to mild	77 (46.7)	54 (43.5)	
Moderate to severe	88 (53.3)	70 (56.5)	
Compression fracture	25 (15.2)	12 (9.7)	0.168
Spondylolisthesis	36 (21.8)	30 (24.2)	0.634
Epidural approach, *n*			0.778
Interlaminar	14 (8.5)	8 (6.5)	
Transforaminal	127 (77.0)	96 (77.4)	
Caudal	24 (14.5)	20 (16.1)	
Frailty status			0.003
Robust	57 (34.5)	30 (24.2)	
Prefrail	84 (50.9)	58 (46.8)	
Frail	24 (14.5)	36 (29.0)	

*Note:* Values are presented as the mean ± standard deviation, median (interquartile range), or number of patients (%). Medical comorbidities: hypertension, diabetes mellitus, and cardiovascular disease.

Abbreviations: BMI, body mass index; ESI, epidural steroid injection; MRI, magnetic resonance imaging; NRS, numeric rating scale.

**Table 2:** Frailty status in patients with good and poor analgesia following ESI according to sex.

	**Males**				**Females**			
	**Robust (*n* = 37)**	**Prefrail (*n* = 45)**	**Frail (*n* = 26)**	***p* value**	**Robust (*n* = 50)**	**Prefrail (*n* = 97)**	**Frail (*n* = 34)**	***p* value**
Good analgesia	24 (64.9)	26 (57.8)	11 (42.3)	0.083	33 (66.0)	58 (59.8)	13 (38.2)	0.016
Poor analgesia	13 (35.1)	19 (42.2)	15 (57.7)		17 (34.0)	39 (40.2)	21 (61.8)	

*Note:* Values are presented as the mean ± standard deviation.

Abbreviation: ESI, epidural steroid injection.

**Table 3:** Factors associated with poor analgesia after lumbar ESI, as shown by multivariable logistic regression analyses.

**Variables**		**aOR**	**95% CI**	***p* value**
HGS, kg		0.959	0.933–0.985	0.002

Frailty status	Robust	1.000		
	Prefrail	1.293	0.736–2.272	0.372
	Frail	2.673	1.338–5.342	0.005

Abbreviations: aOR, adjusted odds ratio; CI, confidence interval; ESI, epidural steroid injection; HGS, handgrip strength.

## Data Availability

The data that support the findings of this study are available from the corresponding author upon reasonable request.

## References

[1] Park D., Kim H. S. (2022). The Factors Affecting Frailty Among the Elderly in Korea: A Study Using the Frailty Cohort. *International Journal of Environmental Research and Public Health*.

[2] Jacobs J. M., Hammerman-Rozenberg R., Cohen A., Stessman J. (2006). Chronic Back Pain Among the Elderly: Prevalence, Associations, and Predictors. *Spine (Phila Pa 1976)*.

[3] Muraki S., Oka H., Akune T. (2009). Prevalence of Radiographic Lumbar Spondylosis and its Association With Low Back Pain in Elderly Subjects of Population-Based Cohorts: The ROAD Study. *Annals of the Rheumatic Diseases*.

[4] Manchikanti L., Knezevic N. N., Navani A. (2021). Epidural Interventions in the Management of Chronic Spinal Pain: American Society of Interventional Pain Physicians (ASIPP) Comprehensive Evidence-Based Guidelines. *Pain Physician*.

[5] Kang W. Y., Lee J. W., Lee E., Kang Y., Ahn J. M., Kang H. S. (2019). Systemic Effects of Fluoroscopically Guided Epidural Steroid Injection With Dexamethasone. *The Korean Journal of Pain*.

[6] Benny B. V., Patel M. Y. (2014). Predicting Epidural Steroid Injections With Laboratory Markers and Imaging Techniques. *The Spine Journal*.

[7] Paidin M., Hansen P., McFadden M., Kendall R. (2011). Contrast Dispersal Patterns as a Predictor of Clinical Outcome With Transforaminal Epidural Steroid Injection for Lumbar Radiculopathy. *Pharmacy Management R*.

[8] Kim S. H., Park S. J., Yoon K. B., Jun E. K., Cho J., Kim H. J. (2022). Influence of Handgrip Strength and Psoas Muscle Index on Analgesic Efficacy of Epidural Steroid Injection in Patients With Degenerative Lumbar Spinal Disease. *Pain Physician*.

[9] Cruz-Jentoft A. J., Bahat G., Bauer J. (2019). Sarcopenia: Revised European Consensus on Definition and Diagnosis. *Age and Ageing*.

[10] Clegg A., Young J., Iliffe S., Rikkert M. O., Rockwood K. (2013). Frailty in Elderly People. *The Lancet*.

[11] Hoogendijk E. O., Afilalo J., Ensrud K. E., Kowal P., Onder G., Fried L. P. (2019). Frailty: Implications for Clinical Practice and Public Health. *The Lancet*.

[12] Moskven E., Bourassa-Moreau É., Charest-Morin R., Flexman A., Street J. (2018). The Impact of Frailty and Sarcopenia on Postoperative Outcomes in Adult Spine Surgery. A Systematic Review of the Literature. *The Spine Journal*.

[13] Flexman A. M., Street J., Charest-Morin R. (2019). The Impact of Frailty and Sarcopenia on Patient Outcomes After Complex Spine Surgery. *Current Opinion in Anaesthesiology*.

[14] Kim S., Kim M., Jung H. W., Won C. W. (2020). Development of a Frailty Phenotype Questionnaire for Use in Screening Community-Dwelling Older Adults. *Journal of the American Medical Directors Association*.

[15] Mandell J. C., Czuczman G. J., Gaviola G. C., Ghazikhanian V., Cho C. H. (2017). The Lumbar Neural Foramen and Transforaminal Epidural Steroid Injections: An Anatomic Review With Key Safety Considerations in Planning the Percutaneous Approach. *American Journal of Roentgenology*.

[16] Chang M. C., Lee D. G. (2018). Outcome of Transforaminal Epidural Steroid Injection According to the Severity of Lumbar Foraminal Spinal Stenosis. *Pain Physician*.

[17] Guen Y. L., Joon W. L., Hee S. C., Kyoung-Jin O., Heung S. K. (2011). A New Grading System of Lumbar Central Canal Stenosis on MRI: An Easy and Reliable Method. *Skeletal Radiology*.

[18] Dworkin R. H., Turk D. C., Wyrwich K. W (2008). Interpreting the Clinical Importance of Treatment Outcomes in Chronic Pain Clinical Trials: IMMPACT Recommendations. *The Journal of Pain*.

[19] Angulo J., El Assar M., Álvarez-Bustos A., Rodríguez-Mañas L. (2020). Physical Activity and Exercise: Strategies to Manage Frailty. *Redox Biology*.

[20] Wilson D., Jackson T., Sapey E., Lord J. M. (2017). Frailty and Sarcopenia: The Potential Role of an Aged Immune System. *Ageing Research Reviews*.

[21] Fried L. P., Tangen C. M., Walston J. (2001). Frailty in Older Adults: Evidence for a Phenotype. *The Journals of Gerontology Series A: Biological Sciences and Medical Sciences*.

[22] Coyle P. C., Sions J. M., Velasco T., Hicks G. E. (2015). Older Adults With Chronic Low Back Pain: A Clinical Population Vulnerable to Frailty?. *Journal of Frailty & Aging*.

[23] Leopoldino A. A. O., Megale R. Z., Diz J. B. M (2021). Influence of Frailty Status on Pain, Disability, and Quality of Life in Older Adults With Acute Low Back Pain: Results From the Back Complaints in the Elders (BACE-Brazil) Study. *Canadian Journal on Aging*.

[24] Esses G. J., Liu X., Lin H. M., Khelemsky Y., Deiner S. (2019). Preoperative Frailty and its Association With Postsurgical Pain in an Older Patient Cohort. *Regional Anesthesia and Pain Medicine*.

[25] Flexman A. M., Charest-Morin R., Stobart L., Street J., Ryerson C. J. (2016). Frailty and Postoperative Outcomes in Patients Undergoing Surgery for Degenerative Spine Disease. *The Spine Journal*.

[26] Park C., Ko F. C. (2021). The Science of Frailty: Sex Differences. *Clinics in Geriatric Medicine*.

[27] Zhang Q., Guo H., Gu H., Zhao X. (2018). Gender-Associated Factors for Frailty and Their Impact on Hospitalization and Mortality Among Community-Dwelling Older Adults: A Cross-Sectional Population-Based Study. *PeerJ*.

[28] Kim H. J., Rho M., Yoon K. B., Jo M., Lee D. W., Kim S. H. (2022). Influence of Cross-Sectional Area and Fat Infiltration of Paraspinal Muscles on Analgesic Efficacy of Epidural Steroid Injection in Elderly Patients. *Pain Practice*.

[29] Gheller B. J., Riddle E. S., Lem M. R., Thalacker-Mercer A. E. (2016). Understanding Age-Related Changes in Skeletal Muscle Metabolism: Differences Between Females and Males. *Annual Review of Nutrition*.

[30] Geisler C., Braun W., Pourhassan M. (2016). Gender-Specific Associations in Age-Related Changes in Resting Energy Expenditure (REE) and MRI Measured Body Composition in Healthy Caucasians. *The Journals of Gerontology Series A: Biological Sciences and Medical Sciences*.

[31] Hoogendijk E. O., Dent E. (2022). Trajectories, Transitions, and Trends in Frailty Among Older Adults: A Review. *Annals of Geriatric Medicine and Research*.

[32] Gill T. M., Gahbauer E. A., Allore H. G., Han L. (2006). Transitions Between Frailty States Among Community-Living Older Persons. *Archives of Internal Medicine*.

[33] Romero-Ortuno R., Hartley P., Davis J. (2021). Transitions in Frailty Phenotype States and Components Over 8 years: Evidence From the Irish Longitudinal Study on Ageing. *Archives of Gerontology and Geriatrics*.

